# Therapeutic Approaches in Modulating the Inflammatory and Immunological Response in Patients With Sepsis, Acute Respiratory Distress Syndrome, and Pancreatitis: An Expert Opinion Review

**DOI:** 10.7759/cureus.18393

**Published:** 2021-09-30

**Authors:** Yatin Mehta, Subhal B Dixit, Kapil Zirpe, Randhir Sud, Palepu B Gopal, Parvaiz A Koul, Vijay K Mishra, Abdul S Ansari, Vijay S Chamle

**Affiliations:** 1 Institute of Critical Care and Anesthesiology, Medanta - The Medicity, Gurugram, IND; 2 Critical Care Medicine, Sanjeevan Hospital, Pune, IND; 3 Neurocritical Care, Grant Medical Foundation, Ruby Hall Clinic, Pune, IND; 4 Institute of Digestive & Hepatobiliary Sciences, Medanta - The Medicity, Gurugram, IND; 5 Department of Critical Care, Continental Hospitals, Hyderabad, IND; 6 Department of Pulmonary Medicine, Sher-i-Kashmir Institute of Medical Sciences, Srinagar, IND; 7 Medica Institute of Critical Care, Bhagwan Mahavir Medica Superspecialty Hospital, Ranchi, IND; 8 Department of Critical Care Services, Nanavati Super Specialty Hospital, Mumbai, IND; 9 Medical Affairs, Urihk Pharmaceuticals, Mumbai, IND

**Keywords:** immunomodulators, sepsis, ards, covid-19, steroid, ulinastatin

## Abstract

Immunomodulation has long been an adjunct approach in treating critically ill patients with sepsis, acute respiratory distress syndrome (ARDS), and acute pancreatitis (AP). Hyperactive immune response with immunopathogenesis leads to organ dysfunction and alters the clinical outcomes in critically ill. Though the immune response in the critically ill might have been overlooked, it has gathered greater attention during this novel coronavirus disease 2019 (COVID-19) pandemic. Modulating hyperactive immune response, the cytokine storm, especially with steroids, has shown to improve the outcomes in COVID-19 patients. In this review, we find that immune response pathogenesis in critically ill patients with sepsis, ARDS, and AP is nearly similar. The use of immunomodulators such as steroids, broad-spectrum serine protease inhibitors such as ulinastatin, thymosin alpha, intravenous immunoglobulins, and therapies such as CytoSorb and therapeutic plasma exchange may help in improving the clinical outcomes in these conditions. As the experience of the majority of physicians in using such therapeutics may be limited, we provide our expert comments regarding immunomodulation to optimize outcomes in patients with sepsis/septic shock, ARDS, and AP.

## Introduction and background

Immune dysfunction is common in critically ill patients who are more vulnerable to infections and the systemic consequences of dysfunctional defense responses. Immune dysfunction directly affects patient morbidity and mortality [1-3]. Critically ill patients respond differently to the injury. The response may be characterized by either pronounced inflammatory reaction or injury-related immunosuppression [2,4]. The exaggerated immune response is primarily due to the release of proinflammatory cytokines, often referred to as cytokine storm, which is common in critically ill patients with infections or injuries (e.g., sepsis, acute respiratory distress syndrome [ARDS]) [5]. Even in patients with acute pancreatitis (AP), the early systemic inflammatory response is associated with severe disease, and systemic inflammation and organ failure depict the same mechanisms observed in sepsis and ARDS [6,7]. The progression of dysregulated immune response along with infection, inflammation, ischemia, and/or shock leads to multiorgan dysfunction (MODS) and ultimately results in death [8].

Encouraging evidence suggests that modulating the immune response in critically ill patients can be a different therapeutic approach. Metanalyses in patients with severe sepsis or septic shock have reported a significant reduction in all-cause mortality, reduced incidence of MODS, and reduced duration of mechanical ventilation with ulinastatin and with a combination of thymosin alpha-1 (Tα1) and ulinastatin [9,10]. In patients with ARDS, the use of agents such as steroids [11,12] and ulinastatin [13] has been shown to reduce mortality and duration of mechanical ventilation significantly. Although some reports suggested possible benefits in terms of reduced length of hospital stay, the need for surgical intervention, and the mortality rate with the use of steroid therapy in severe AP [14], the role of steroids in AP is controversial. Ulinastatin showed a significant effect on inflammatory markers such as C-reactive protein (CRP), interleukin-6 (IL-6), and tumor necrosis factor-alpha (TNF-α), as well as reduced and prevented MODS and lowered mortality in AP [15,16]. However, lack of mortality reduction benefits with some immunomodulatory agents, such as drotrecogin alpha in septic shock [17] and tocilizumab in novel coronavirus disease 2019 (COVID-19) [18], has raised certain questions regarding the use of immunomodulators in critically ill patients. Therefore, we need to better understand the possible utility of immunomodulating agents for critically ill patients. In this review, we discuss the critical aspects regarding the use of immunomodulators in the management of critically ill patients with sepsis, ARDS, and AP.

Approach to the development of expert opinions

Developing an expert opinion document was first conceptualized by a nationally known specialist intensivist. Along with the involvement of other intensivists, pulmonologists, and gastroenterologists, five core expert group meetings were conducted on an online platform under the aegis of the Sepsis Forum of India (SFI). One expert from the respective fields discussed the aspects of immunological alterations and possible immunomodulatory therapeutic approaches in the management of sepsis, ARDS, and AP. After collating the discussion from all meetings, the expert opinions were formulated. Overall, 52 experts in five meetings provided their valuable suggestions to finalize the expert opinions.

## Review

Pathophysiology of immune dysfunction

Sepsis

**Figure 1:**
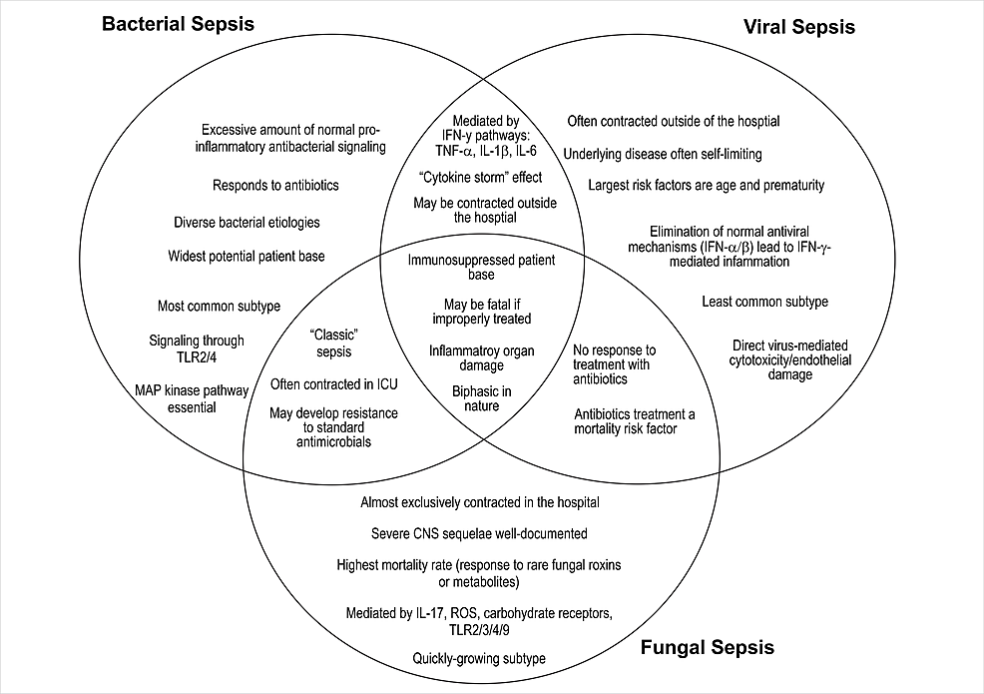
A pictorial representation of the similarities and differences in mechanism, circumstances, and patient base between the three major types of pathogenic sepsis. The best-known mechanisms for each sepsis type are represented, although these molecules and mechanisms are not universal. CNS: central nervous system; IFN: interferon; IL: interleukin; ICU: intensive care unit; MAP: mitogen-activated protein; ROS: reactive oxygen species; TLR: toll-like receptor; TNF: tumor necrosis factor Dolin HH, Papadimos TJ, Chen X, Pan ZK: Characterization of pathogenic sepsis etiologies and patient profiles: a novel approach to triage and treatment. Microbiol Insights. 2019, 12:1178636118825081. [19] (Reprinted with permission from SAGE publications).

Broadly, in any type of sepsis (e.g., bacterial, viral, fungal), invasion by a pathogen, cellular signaling, and inflammatory response leading to organ dysfunction are evident. The severity of changes may vary in individuals depending on multiple factors, with host factors being the primary factor. However, subtle differences in each type of sepsis may also contribute to different levels of organ dysfunction. Such differentiation might help in selecting the appropriate immunomodulatory therapy [19]. Here, we briefly discuss the differences in various types of sepsis (Figure 1).

In intracellular bacterial infection, cell-mediated immunity plays an important role. Some patients may develop heightened inflammatory responses after initiation of treatment in bacterial infections. This is accompanied by increased levels of circulating IL-1, IL-6, IL-8, IL-10, and TNF-α. In extracellular bacterial infection, along with innate cellular immunity, complement system activation occurs through an alternative pathway and the production of chemokines and cytokines. Cytokines such as TNF-α, IL-1, and IL-6 are produced during the early phase of the infection. These may provide a protective response against bacterial infection. However, exaggerated inflammatory response or the difficulty in controlling the initial response may provoke tissue damage. Lipopolysaccharides (LPS) present in the bacterial wall stimulate an exacerbated production of proinflammatory cytokines leading to hypotension, poor tissue perfusion, and cellular death [20]. Experimental evidence indicates that inhibiting the production of IL-12, interferon-gamma (IFN-γ), and TNF-α with the combination of IL-10 and LPS is protective in septic shock [21].

In viral infections, the virus is phagocytosed by macrophages, dendritic cells, and other phagocytes. IFN-γ and IL-12 stimulate T-cell differentiation into Th1 cells and CD8 T-cells. These cells result in the apoptosis of infected cells and increased production of reactive oxygen species in phagocytes, thereby killing the phagocytosed virus. Antibody production helps in opsonization, increased phagocytosis, and viral clearing. When an aberrant immune response occurs in a viral infection, phagocytosed viral cells break down and present the virus to antigen-presenting cells (APCs). Stimulation of type 2 immunological response (e.g., IL-4, IL-6, IL-13, IL-15, IL-17) results in inflammatory response without viral clearance. Viral replication continues with immune exhaustion. This phase is characterized by increased levels of cytotoxic T-lymphocyte-associated antigen 4, programmed death 1, IFN-γ, and TNF-α. Rather than the initial hyperimmune response, the immunosuppression resulting from viral sepsis is a more important contributor to secondary nosocomial infections and mortality [22].

Fungal infections may be self-limiting as host defense response usually protects against fungi. However, individuals with neutropenia or frequent cellular immune deficiency may develop recurrent mycoses that may sometimes progress to severe forms [21]. Some studies have indicated that modulating immunological responses (such as CD8+ T-cell therapy) may be helpful in invasive fungal infections [23].

The immune response in helminth infections is predominantly antibody-mediated pathogen killing. In various severe protozoal infections, there is evidence that an exacerbated immune response responsible for tissue damage is specific (such as neutrophil action in amebiasis, CD4+ and CD8+ cell-mediated response in Chagas disease, and increased TNF-α and nitric oxide in cerebral malaria) [20].

Acute Respiratory Distress Syndrome

ARDS is usually secondary to primary insults. Inflammatory stimuli from extrapulmonary sites, such as in sepsis (either bacterial or viral), trauma, and transfusion, or locally from the lungs, such as mitochondrial damage-associated molecular patterns (DAMPs), activate macrophages in the alveoli. These release proinflammatory cytokines such as IL-1-beta, TNF-α, IL-6, IL-8, etc., which further recruit macrophages and neutrophils. Excessive and persistent inflammation damage the alveolar epithelium and endothelium. Epithelial damage causes reduced surfactant protein and the functioning of ion channels such as Na⁺/K⁺-ATPase. This then results in decreased fluid clearance. Impaired vascular barrier from endothelial damage increases alveolar permeability. Both these processes cause alveolar edema that interferes with alveolar gas exchange leading to hypo-oxygenation. ARDS patients have elevated inflammatory cytokines such as IL-1β, TNF-α, IL-6, and IL-8 in bronchoalveolar lavage fluid and plasma. In patients with nonresolving ARDS, systemic immunosuppression may be observed along with persistent pulmonary inflammation [24].

Acute Pancreatitis

External factors (e.g., alcohol consumption) cause hyperstimulation of cholecystokinin receptor (CCKR). It leads to dysregulation of intracellular acinar proteases and the generation of trypsin from trypsinogen causing acinar cell injury and the release of DAMPs. These act on toll-like receptors (TLRs) of APCs that produce a mixture of proinflammatory cytokines such as IL-6, IL-1β, and TNF-α. Neutrophils migrate into the pancreas and increase trypsinogen activation and induce acinar cell damage, directly or via the production of neutrophil extracellular traps. The release of proinflammatory cytokines alters the interstitial permeability. Furthermore, translocation of gut microflora into the circulation is also observed. These act via TLRs and nucleotide-binding oligomerization domain (NOD) to activate acinar cells. The stimuli via CCKR and NOD signaling lead to the activation of nuclear factor-kappa B, TNF-α, and type I IFN factors that sustain the inflammation in the pancreas [25].

Immunomodulators in sepsis

In the management of sepsis, the primary aim of immunomodulatory therapies is to reinstate a balanced immune response to infection by reducing inflammation and repairing immune refractoriness [26]. In addition to mainstay therapies such as antibiotics, fluids, and/or vasopressors, various immunomodulators can be helpful in sepsis and septic shock.

Steroids

By virtue of their action, steroids can help limit the proinflammatory response and preserve innate immunity. Over the past four decades, steroid use in sepsis and septic shock has been controversial. Various meta-analyses have reported conflicting results for mortality benefits [27]. The recent Adjunctive Corticosteroid Treatment in Critically Ill Patients With Septic Shock (ADRENAL) trial showed no difference in 90-day mortality with continuous infusion of hydrocortisone (at a dose of 200 mg per day) or placebo for seven days. However, the median time to resolution of shock, discharge from the intensive care unit (ICU), and the cessation of mechanical ventilation were significantly shorter in the hydrocortisone group, with no difference in the rate of complications (e.g., infections, wound dehiscence) [28]. A recent pooled analysis of 16 meta-analyses established that a long course of low-dose corticosteroids in sepsis and septic shock is associated with a significant reduction in 28-day mortality, in-hospital deaths, and ICU deaths, as well as reduced length of stay in the ICU [29]. Another meta-analysis of 42 randomized controlled trials (RCTs) demonstrated that corticosteroids might achieve a small reduction in 28-day mortality and higher rates of shock reversal at day seven [30]. The rapid recommendation from the British Medical Journal (BMJ) suggests the use of steroids in all patients with sepsis. They also recommend no meaningful differences in different patient categories such as septic shock, pneumonia, ARDS, or other sources of sepsis, or those who were sicker. Based on evidence from RCTs, a study advised hydrocortisone 200-300 mg/day, given either as an infusion or as boluses every six hours for seven to fourteen days [31]. These recommendations, however, do not differentiate between bacterial or viral sepsis and are probably for the treatment of bacterial sepsis. In viral sepsis, steroid use has been controversial. In a meta-analysis of 19 studies with 4,916 patients suffering from influenza, steroid treatment was associated with increased mortality and nosocomial infections. Moreover, the duration of mechanical ventilation and ICU stay was prolonged with steroid use [32]. Additionally, steroids were found to be of no benefit in the treatment of severe acute respiratory syndrome and Middle East respiratory syndrome (MERS) [33,34]. However, in COVID-19 patients, dexamethasone (6 mg once daily for 10 days) in addition to the usual care among hospitalized patients was associated with a 17% relative risk reduction in 28-day mortality compared to the usual care alone. The mortality benefit was more pronounced in patients receiving invasive mechanical ventilation (36% relative risk reduction) and those receiving oxygen without invasive mechanical ventilation (18% relative risk reduction) [35]. Similarly, studies found the utility of steroid treatment in dengue infection including dengue shock syndrome [36,37]. These observations raise essential concerns such as the optimal dose and timing of administration of steroids in viral sepsis. Further evidence is required to advise steroids in other viral illnesses. The role of steroids in fungal and parasitic sepsis needs to be evaluated further.

Ulinastatin

Ulinastatin is a broad-spectrum serine protease inhibitor. Serine proteases are involved in systemic inflammation and cell apoptosis [37,38]. Ulinastatin inhibits proteases such as trypsin, chymotrypsin, thrombin, kallikrein, neutrophil elastase, and cathepsin. It results in the regulation of inflammatory response in the form of reduction in proinflammatory cytokines such as IL-6, IL-8, IL-4, and TNF-α, as well as a decrease in inflammatory markers such as CRP, with the ultimate reduction in neutrophil infiltration. Simultaneously, there is an increase in IL-10, which downregulates the Th-1 response. Furthermore, it inhibits apoptosis by reducing mitochondrial damage via its antioxidant actions. In addition, ulinastatin normalizes coagulation disturbances, decreases endothelial dysfunction, improves tissue perfusion, and restores organ function [9,39,40]. A meta-analysis of 13 RCTs involving 1,358 patients with sepsis, severe sepsis, or septic shock observed a significant reduction in mortality and incidence of MODS. There was a significant reduction in the Acute Physiologic Assessment and Chronic Health Evaluation II (APACHE-II) score indicating that the administration of ulinastatin reduced disease severity. Significant effect on the reduction of IL-6 and serum TNF-α, as well as an increase in IL-10, indicates excellent anti-inflammatory activity with ulinastatin [9]. It is also effective in sepsis among critically ill patients [41]. In a recent RCT from India, Yadav et al. compared ulinastatin to hydrocortisone, ascorbic acid, and thiamine (HAT) therapy in patients with sepsis and septic shock. The 28-day mortality rate was significantly lower in the ulinastatin group than in HAT treatment (20% vs. 50%, p = 0.015). The Mean Sequential Organ Failure Assessment (SOFA) score on days 3 and 5 as well as the rate of reduction in procalcitonin levels were significantly greater in the ulinastatin group. Thus, ulinastatin may play a beneficial role in the early management of sepsis and septic shock [42].

In viral sepsis, ulinastatin has been used in some viral infections. Recently, when used in high doses among patients with moderate-to-severe COVID-19, ulinastatin was associated with improvement in clinical parameters, inflammatory parameters, reduction in oxygen requirements, and varying degree of resorption of pulmonary lesions. No safety concerns were reported [43]. Based on experience, experts from India [44] and China [45] advise using ulinastatin to manage patients hospitalized with COVID-19. Thus, ulinastatin has the potential for altering outcomes in COVID-19 which needs to be evaluated further. In patients with severe dengue, Javeri et al. demonstrated that compared to placebo, ulinastatin (200,000 IU) 12 hourly for five days was associated with lower mortality on day 28 and prevented MODS [46]. Another study from China involving patients with “novel bunyavirus” disease (or severe fever with thrombocytopenia) reported that ulinastatin in combination with immunoglobulin, ribavirin, recombinant granulocyte colony-stimulating factor (GM-CSF), and magnesium isoglycyrrhizinate along with supportive therapy resulted in improved survival in patients with severe disease [47]. In another study from China, Xi et al. used ulinastatin and steroids in treating nearly 30% and 29% of patients with H1N1 influenza, respectively. They reported that low-dose steroids may be used in septic shock and ulinastatin may be an alternative to steroids in patients with comorbidities such as stress ulcers, hyperglycemia, and those at risk of fungal infections [48]. These results demand further evaluation of ulinastatin in severe viral sepsis caused by different viruses.

The direct evidence regarding the use of ulinastatin in fungal sepsis is not available. A retrospective study from China involving 295 patients with sepsis reported fungi cultured in 17.63% samples along with Gram-negative (40.00%) and Gram-positive (26.10%) bacteria. On multivariate analysis, treatment with ulinastatin reduced the 28-day mortality risk by 44%. There was no subgroup analysis by type of sepsis. Nonetheless, the study provided indirect evidence of possible mortality benefits in fungal sepsis [49].

Thymosin alpha-1

Tα1 is a notable immunomodulator. Being a naturally occurring thymic peptide, it acts as an endogenous regulator of both innate and adaptive immune responses. It has a wide range of biological activities, including antitumor to immune-modulating activities [50]. Two meta-analyses of 12 and 10 RCTs identified significantly lower mortality in critically ill septic patients treated with Tα1-based immunotherapy [51,52]. However, there was no significant effect on the length of ICU stay, the incidence of MODS, and the duration of mechanical ventilation [52]. In combination with immunoglobulin, Tα1 has been shown to lower disease severity, reduce the time on mechanical ventilation, and improve the survival and prognosis in patients with severe sepsis [53]. The combination of ulinastatin and Tα1 reduces inflammatory cytokine response, improves disease severity, and reduces 28-day mortality [10].

Recent studies in critically ill patients with COVID-19 showed conflicting results. Sun et al. reported no difference in 28-day mortality after propensity score matching comparison of Tα1 with controls [54]. In another study, Wu et al. reported significantly lower 28-day mortality in the adjusted model (p = 0.016) with Tα1 than those treated without Tα1 [55]. In another study with severe COVID-19 patients, Tα1 treatment significantly reduced mortality than no treatment (11.11% vs. 30.00%). The benefit was greater in patients with counts of CD8+ T-cells or CD4+ T-cells less than 400/μL or 650/μL, respectively. It indicates that Tα1 reversed T-cell exhaustion and recovered immune reconstitution in COVID-19 [56]. In addition to COVID-19, Tα1 has shown potential benefits in treating viral infections such as hepatitis C, hepatitis B, and human immunodeficiency virus, and has also been used as an influenza vaccine adjuvant [50].

In fungal sepsis, Tα1 may also be effective in providing a positive immunomodulatory response. In *Aspergillosis fumigatus* infection, Tα1 induced functional maturation and IL-12 production by fungus-pulsed dendritic cells [57]. The Efficacy of Thymosin Alpha 1 for Severe Sepsis (ETASS) trial compared Tα1 with the control population. Pure fungal sepsis was reported in 11.6% and 12.2% of patients in two groups, whereas 30.9% and 31.7% had mixed growth on cultures, respectively. Though not evaluated by the pathogen isolated, the study found lower 28-day mortality with Tα1 treatment (26.0% vs. 35.0%) [58].

Hydrocortisone, Ascorbic Acid, and Thiamine

Regarding HAT combination therapy in sepsis and septic shock, initial retrospective studies reported benefits in mortality [59]. However, recent studies disproved their benefits in reducing mortality [60,61]. In a recent study from India, Wani et al. observed no difference in in-hospital and 30-day mortality with the use of HAT therapy in sepsis [62]. Shi et al. pooled data from four RCTs and observed no mortality benefits but a significant reduction in SOFA score and time of vasopressor use. In contrast, pooled data from five cohort studies showed a significant decrease in mortality and SOFA score but not the duration of vasopressor use [63]. These findings indicate HAT can still be an adjunct therapy to reduce sepsis severity. Further large-scale RCTs are required to draw more definite conclusions.

Intravenous Immunoglobulins

Treatment with intravenous immunoglobulins (IVIg) has been under consideration for adjunctive treatment of sepsis for the last three to four decades. A meta-analysis of 14 RCTs performed a decade ago showed a significant reduction in mortality with IVIg treatment in patients with sepsis. However, the benefit diminished when only high-quality studies were pooled. The authors observed substantial heterogeneity in studies [64]. Two recent meta-analyses also demonstrated mortality reduction benefits with IVIg in patients with sepsis. However, there was heterogeneity in the studies [65,66]. The optimal dose identified to provide greater benefits was 1.5-2 g/kg [66]. These data indicate the possible adjunctive role of IVIg in patients with sepsis.

IVIg has been used to treat chronic parvovirus infections complicated by anemia. In patients with severe acute respiratory syndrome coronavirus (SARS-CoV) infection, IVIg improved leukocyte and platelet counts. In combination with steroids, IVIg has also been shown to enhance the recovery of SARS-CoV patients [67]. In a review of retrospective and open-label studies, Galeotti et al. observed that IVIg immunotherapy could benefit severe and critically ill COVID‐19 patients [68]. A multicenter retrospective study from China showed a reduction in 28-day mortality in severe COVID-19 patients after treatment with high-dose IVIg [69]. A double-blind RCT by Gharebaghi et al. demonstrated that IVIg therapy independently reduced in-hospital mortality in severe COVID-19 patients [70]. Though limited by a small sample, this RCT provided encouraging evidence for the use of IVIg in COVID-19. IVIg administration within 48 hours of admission was associated with lower mortality (23.3 vs. 57.1%) [71].

Granulocyte-macrophage Colony-Stimulating Factor

In patients with sepsis, GM-CSF improved recovery from infection, decreased hospital length of stay, decreased days requiring mechanical ventilation, and decreased medical costs [72]. In an RCT from Meisel et al. involving patients with severe sepsis or septic shock and sepsis-associated immunosuppression, GM-CSF treatment normalized monocytic human leukocyte antigen-DR in all patients (n = 19) compared to only three of nineteen patients in the control group (p < 0.001). There was a significantly lower APACHE-II score and a nonsignificantly shorter hospital and ICU stay with GM-CSF treatment. Thus, GM-CSF is shown to reverse sepsis-induced immunosuppression [73].

In a viral infection, if administered in the early phase of the disease, GM-CSF can be helpful. With an assumption of the possible benefit of GM-CSF in COVID-19, a trial is underway with sargramostim, a recombinant humanized GM-CSF, in COVID-19 patients with acute hypoxic respiratory failure (ClinicalTrials.gov Identifier: NCT04326920).

Other Therapies

Extracorporeal cytokine removal (CytoSorb®) use is effective and safe in patients with septic shock. Initiation within 24 to 48 hours of the onset of sepsis is associated with better clinical outcomes [74,75]. However, further research is required to establish its role across the sepsis spectrum. It is being used as an adjunctive treatment in critically ill COVID-19 patients. Some reports indicate positive findings with its usage [76]. An open-label study is underway in such patients to further establish the efficacy and safety of CytoSorb in COVID-19 [77]. Therapeutic plasma exchange (TPE) has been under consideration for sepsis management for more than three decades [78]. Some studies have reported benefits in improving clinical outcomes [79,80]. A small, single-center study showed mortality benefit with TPE in patients with COVID-19 [81]. However, this strategy needs further evaluation in prospective RCTs. Convalescent plasma (CP) use has been suggested in various viral infections, including MERS and H1N1 [82]. In patients with severe COVID-19, multiple studies with a small sample size have shown the possible benefits of CP in improving clinical conditions [83,84]. However, a multicenter, open-label, phase II study, the PLACID trial, from India reported that the use of CP in moderate COVID-19 patients does not reduce progression to severe disease or reduce overall mortality [85]. However, further studies may be necessary to establish the utility of CP in improving clinical outcomes, especially in severe/critically ill COVID-19 patients.

Immunomodulators in acute respiratory distress syndrome

Steroids

Multiple meta-analyses in patients with ARDS observed that steroids reduce mortality, reduce ventilation duration, and increase ventilator-free days [86-88]. A Cochrane meta-analysis observed benefits of 90-day mortality reduction and improved ventilator-free days with steroids compared to placebo [89]. The timing of administration, dose of steroids, and duration of therapy are essential determinants in achieving optimal benefits. Early and low-dose administration is key to improving outcomes [90]. In patients with COVID-19-associated ARDS, a meta-analysis of 44 studies reported beneficial effects on short-term mortality and reduced need for mechanical ventilation [91]. Similar benefits were reported in a meta-analysis from the World Health Organization’s Rapid Evidence Appraisal for COVID-19 Therapies (REACT) Working Group [92]. Late administration of steroids is not associated with any clinical benefits [93].

Ulinastatin

Ulinastatin, via its broad-spectrum serine protease inhibition, modulates inflammatory responses in ARDS. A meta-analysis of 33 RCTs in 2,344 patients with ARDS observed significant reduction in rates of mortality (risk ratio [RR] = 0.51, 95% confidence interval [CI] = 0.43 to 0.61) and ventilator-associated pneumonia (RR = 0.50, 95% CI = 0.36 to 0.69). There was significant reduction in the duration of mechanical ventilation (standard mean difference [SMD] = -1.29, 95% CI = -1.76 to -0.83), duration of ICU stay (SMD = -1.38, 95% CI = -1.95 to -0.80), and hospital stay (SMD = -1.70, 95% CI = -2.63 to −0.77). Furthermore, there was a significant increase in patients’ oxygenation index, reduction in respiratory rate, as well as TNF-α, IL-6, and IL-8 [13]. In addition, it improved antioxidant capacity, as indicated by the levels of superoxide dismutase and malondialdehyde [94]. Thus, in patients with ARDS, ulinastatin should be considered as one of the potential therapeutic options to reduce mortality and derive optimal clinical benefits.

Vitamin C

Vitamin C is a known antioxidant with immunomodulating properties. In the CITRIS-ALI trial involving patients with sepsis and severe acute respiratory failure, vitamin C infusion (50 mg/kg) failed to show the benefits of improving organ dysfunction scores or improving the markers of inflammation and vascular injury. However, the secondary outcome of 28-day mortality was significantly lower in the vitamin C group than in the placebo group [95]. Bharara et al. reported the beneficial effect of high-dose vitamin C in a case of recurrent ARDS [96]. A meta-analysis of five studies involving critically ill patients, administration of vitamin C showed vasopressor sparing effect and reduced need for mechanical ventilation but without reducing mortality [97].

In patients with COVID-19-associated ARDS, low levels of vitamin C have been reported. Although the etiopathogenesis of low vitamin C levels is not clear, this could be due to increased metabolic consumption secondary to heightened inflammatory response, increased filtration via kidneys (glomerular hyperfiltration), or decreased absorption from the gastrointestinal tract [98]. A pilot trial in critically ill COVID-19 patients observed that high-dose vitamin C (12 g per 50 mL every 12 hours for seven days) in comparison to placebo did not improve 28-day mortality but did improve invasive mechanical ventilation-free days [99]. Thus, there is a need to generate further evidence to establish the role of high-dose vitamin C in improving hard endpoints such as mortality in patients with ARDS.

Interleukin 6 Inhibitor: Tocilizumab

Multiple studies have reported the possible benefits of tocilizumab in COVID-19 patients with ARDS [100,101]. COVACTA, a phase 3, multicenter RCT, failed to show the benefits of 28-day mortality reduction or clinical improvement over that of placebo [102]. However, in a recent RECOVERY trial involving patients with hypoxia and evidence of systemic inflammation, the addition of tocilizumab to the standard of care was associated with a significantly lower relative risk of 28-day mortality. In patients not receiving mechanical ventilation at baseline, tocilizumab use was associated with a substantially lower rate of a composite outcome of invasive mechanical ventilation or death [103]. These results are further supported by observation from a retrospective study that reported a significant reduction in the risk of invasive mechanical ventilation or death in patients with severe COVID-19 pneumonia treated with tocilizumab [104].

Other Therapies

An anti-CD6 monoclonal antibody, Itolizumab, is a humanized recombinant immunoglobulin G1 monoclonal antibody. It was approved in India for restricted emergency use for cytokine release syndrome in moderate-to-severe ARDS caused by COVID-19 [105]. Multiple clinical studies observed positive results with Itolizumab in different levels of COVID-19 disease severity [106-108]. In severe ARDS, Kumar et al. reported greater mortality and recovery benefits with Itolizumab than control [109]. Carbon monoxide (CO) protects against oxidative stress and cell death and suppresses inflammation. A phase I trial showed benefits of improving lung injury and SOFA score with low-dose inhaled CO [110]. Further studies are required to establish its role in ARDS. Mesenchymal stromal cells are known to have immunomodulatory and pro-reparative effects. Though some encouraging results have been reported by preclinical and phase I studies, further research is needed to establish their definitive role in the management of ARDS [111]. Multiple therapeutic strategies are currently under investigation. A detailed discussion of these therapies is out of the scope of this paper. Horie et al. have reviewed the current investigational approaches in detail [111].

Immunomodulators in acute pancreatitis

Octreotide

Somatostatin, a neuropeptide, is known to exert a significant anti-inflammatory effect in AP. Reduction in the levels of somatostatin along with the concomitant rise in IL-6 and TNF-α is known in the early course of AP. Administration of a somatostatin analog, octreotide, has been shown to reduce the severity of AP, especially in obese patients, with improvement in somatostatin levels and reduction in inflammatory markers such as IL-6. However, conflicting results have been observed in different studies. It may be because of differences in the dosage and timing of administration of octreotide [112,113]. Further prospective RCTs are required to establish the role of octreotide in AP.

Ulinastatin

In addition to its known anti-inflammatory actions with the reduction in CRP, IL-6, and TNF-α [16], ulinastatin has been shown to reduce the inflammatory response and tissue damage by increasing the proportion of T-regulatory (Tregs) cells [114]. Ulinastatin improves existing organ dysfunction and prevents the development of new organ dysfunction, as well as reduces hospitalization and mortality in patients with severe AP [16,115,116]. The dose of ulinastatin may affect the outcome. He et al. observed that compared to 200,000 IU per day, 400,000 and 600,000 IU groups had significantly lower mortality rates [117]. In combination with octreotide, ulinastatin has been shown to exert better anti-inflammatory effects, improve the serum and clinical parameters, and reduce complications and mortality in patients with severe AP [118]. In patients at low-to-average risk of post-endoscopic retrograde cholangiopancreatography (ERCP) pancreatitis (PEP), a meta-analysis observed that the prophylactic administration of ulinastatin at a high dose (150,000 or 200,000 IU) before or during ERCP significantly reduced the risk of PEP [119]. Further studies in patients at high risk of PEP are warranted. Thus, ulinastatin appears to be one of the most effective therapeutic options for the treatment of severe AP to improve clinical outcomes, prevent organ dysfunction, and reduce mortality. Furthermore, it is effective in lowering PEP.

Steroids

Dong et al. performed a meta-analysis of six RCTs involving a total of 430 patients. Compared with no steroids, therapy with corticosteroids was associated with a significantly lower hospital stay, the requirement of surgical intervention, and decreased mortality in patients with severe AP. It was observed that low-dose therapy with a duration ranging from three to fourteen days was the most effective. However, as studies were not blinded, the authors advised consideration of investigator bias [14]. Currently, the role of steroids in the management of AP is largely controversial, and further large-scale, blinded studies are needed to establish their role in any form of AP.

Other Agents

In patients at high risk of PEP, compared to placebo, a single dose of rectal indomethacin administered immediately after ERCP was associated with a significantly lower incidence of PEP (9.2% vs. 16.9%, p = 0.005). Moderate-to-severe pancreatitis was also significantly lower in indomethacin (4.4% vs. 8.8%, p = 0.03) [120]. Multiple target-specific molecules such as anti-TNF antibodies, IL-1 blockers, IL-20 agonists, and endothelin blockers are under investigation for the treatment of AP [6].

Expert opinions

Immune (Inflammatory) Response May be Overlooked in Most Critically Ill Patients

Immune response in critically ill patients is mostly overlooked, possibly because its functional status cannot be adequately assessed [2]. Targeting immune response could be a potential therapeutic option that has been brought into focus by the COVID-19 pandemic [121]. It is crucial to identify immune hyperactivation early in the course of illness.

One Should Consider Immunomodulating Therapy in the Management of Sepsis, Acute Respiratory Distress Syndrome, and Acute Pancreatitis

Immunomodulator therapy should be considered as an adjuvant in the management of critically ill patients with sepsis, ARDS, or AP. However, source control (infection source) is the mainstay of therapy, especially in sepsis patients. In considering immunomodulator therapy, the choice of drug, its dose, and the time of administration are critical aspects. Based on current evidence and our experience, we propose different choices of immunomodulators in sepsis, ARDS, and AP (Table 1). In patients with COVID-19, steroids are preferred immunomodulators. In addition, ulinastatin may also be used when there is predominant involvement of lungs as it has known efficacy in patients with ARDS. Consider Tα1 when lymphocytic suppression is predominant. When there is predominant renal involvement, therapies such as CytoSorb can be considered. In our experience, immunomodulators such as steroids and ulinastatin may be helpful in tropical illnesses. In patients with AP, the early start of an immunomodulator (e.g., ulinastatin) within 72 hours is essential to preventing the development of MODS. Although our experience is limited, the use of immunomodulators such as ulinastatin may be considered in late deterioration of AP (e.g., in the second or third week) as sepsis tends to occur more frequently during this period. Lack of specific guidelines for the use of immunomodulators may restrict their widespread use.

**Table 1:** Choices of different immunomodulating therapies in sepsis, ARDS, and AP. ARDS: acute respiratory distress syndrome; AP: acute pancreatitis; HAT: hydrocortisone, ascorbic acid, thiamine; IVIg: intravenous immunoglobulin Yes: drug advised; No: drug not advised; ?: evidence or experience is limited ^#^Only in septic shock; ^a^ARDS may be from bacterial or viral sepsis, AP, or other etiologies of acute lung injury; *currently molecules may not be approved for use in specific indications. However, evidence is supportive of their use.

Condition	Sepsis/septic shock	ARDS^a^	AP
Steroids	Yes^#^	Yes	No
Ulinastatin	Yes	Yes*	Yes
Thymosin alpha-1	Yes*	?	No
HAT therapy	?	No	No
High-dose Vitamin C	?	?	No
Tocilizumab	No	?	No
IVIg	Yes	No	No
Octreotide	No	No	?

Biomarkers and Disease Severity Criteria Can Assist in Deciding When to Start Immunomodulators

Evidence indicates that early use of immunomodulators, biomarkers, and disease severity scores can assist decision-making. Biomarkers such as procalcitonin (PCT) and CRP are helpful in bacterial sepsis, and CRP, ferritin, and IL-6 may be more useful in viral sepsis. In contrast, β-d-glucan, galactomannan, PCT, and CRP can be used to assess fungal infections. In patients with AP, disease severity scores such as APACHE-II may be more valuable than specific biomarkers. In a resource-limited setting, clinical decision-making should play an important role. Therefore, immunomodulators should be started at the first sign of the development of organ dysfunction.

Timing and Dosage of Administration of Immunomodulators

For sepsis, ARDS, and AP, the use of available immunomodulators at the right dose and at the right time is crucial to derive optimal clinical benefits. A low dose of steroids such as 6 mg of dexamethasone (equivalent to 160 mg, 40 mg, 32 mg of hydrocortisone, prednisone, methylprednisolone, respectively) once daily has been advised in the management of moderate-to-severe COVID-19 patients. The use of ulinastatin in the management of sepsis, ARDS, and AP should be early in the course of disease (e.g., with evidence of organ dysfunction or rising biomarker levels) to derive optimal benefits. A dose of 200,000 IU three times daily for five to seven days has been advised to manage critically ill patients. Tα1 should be initiated early in the course of sepsis. The recommended dose is 1.6 mg twice daily subcutaneously for five to seven days, followed by once per day for one to two days. It may particularly be used in patients with low absolute lymphocyte counts. In patients with severe AP, octreotide may be initiated at a dose of 100 to 500 μg three times daily either subcutaneously or 25 to 50 μg/hour for five to seven days via intravenous infusion.

Future directions

Though not explicitly known at this moment, it is essential to distinguish which immunomodulator may be more specifically useful according to the type of sepsis (bacterial, viral, fungal). Gaining more experience with immunomodulators during the COVID-19 pandemic, we understand that there is a need for prospective studies comparing different immunomodulators in different patient populations. The timing of administration has been a critical aspect in sepsis and ARDS. Further research should focus on exploring this to consolidate the evidence. The utility of various immunomodulators in patients with AP needs to be further explored in prospective studies.

## Conclusions

Hyperactive immune response with immunopathogenesis is established in the progression of sepsis, ARDS, and AP. Although it has often been overlooked in critically ill patients, COVID-19 has brought it into focus. Current evidence indicates that in patients with sepsis and ARDS, immunomodulators such as steroids, ulinastatin, Tα1 have a potential role in improving clinical outcomes. The proven role of ulinastatin in AP including PEP necessitates its early use in preventing MODS. Choosing a specific immunomodulator in a specific patient population such as bacterial or viral sepsis needs to be studied further to determine the effective agent in specific settings. Early administration of immunomodulators at the first sign of organ dysfunction or increasing biomarker levels should be considered to derive optimal outcomes. With the evolution of immunomodulatory therapies such as steroids proving benefits in the COVID-19 pandemic, there is an urgent need to escalate research in assessing various immunomodulators to combat this pandemic effectively and lay the ground for further extension of these therapies in various tropical illnesses as well. In conclusion, we suggest that immunomodulators should be considered adjuncts in sepsis and ARDS and can be the initial choice in severe AP to improve clinical outcomes.

## References

[1] Surbatovic M, Vojvodic D, Khan W (2018). Immune response in critically ill patients. Mediators Inflamm.

[2] Pfortmueller CA, Meisel C, Fux M, Schefold JC (2017). Assessment of immune organ dysfunction in critical illness: utility of innate immune response markers. Intensive Care Med Exp.

[3] Marshall JC, Charbonney E, Gonzalez PD (2008). The immune system in critical illness. Clin Chest Med.

[4] Oberholzer A, Oberholzer C, Moldawer LL (2000). Cytokine signaling--regulation of the immune response in normal and critically ill states. Crit Care Med.

[5] Muszynski JA, Thakkar R, Hall MW (2016). Inflammation and innate immune function in critical illness. Curr Opin Pediatr.

[6] Kylänpää L, Rakonczay Z Jr, O'Reilly DA (2012). The clinical course of acute pancreatitis and the inflammatory mediators that drive it. Int J Inflam.

[7] Singh VK, Wu BU, Bollen TL, Repas K, Maurer R, Mortele KJ, Banks PA (2009). Early systemic inflammatory response syndrome is associated with severe acute pancreatitis. Clin Gastroenterol Hepatol.

[8] Secor VH (1994). The inflammatory/immune response in critical illness: role of the systemic inflammatory response syndrome. Crit Care Nurs Clin North Am.

[9] Wang H, Liu B, Tang Y (2019). Improvement of sepsis prognosis by ulinastatin: a systematic review and meta-analysis of randomized controlled trials. Front Pharmacol.

[10] Liu D, Yu Z, Yin J (2017). Effect of ulinastatin combined with thymosin alpha1 on sepsis: a systematic review and meta-analysis of Chinese and Indian patients. J Crit Care.

[11] Junhai Z, Bangchuan H, Shijin G, Jing Y, Li L (2021). Glucocorticoids for acute respiratory distress syndrome: a systematic review with meta-analysis and trial sequential analysis. Eur J Clin Invest.

[12] Hirano Y, Madokoro S, Kondo Y, Okamoto K, Tanaka H (2020). Corticosteroid treatment for early acute respiratory distress syndrome: a systematic review and meta-analysis of randomized trials. J Intensive Care.

[13] Zhang X, Zhu Z, Jiao W, Liu W, Liu F, Zhu X (2019). Ulinastatin treatment for acute respiratory distress syndrome in China: a meta-analysis of randomized controlled trials. BMC Pulm Med.

[14] Dong LH, Liu ZM, Wang SJ, Zhao SJ, Zhang D, Chen Y, Wang YS (2015). Corticosteroid therapy for severe acute pancreatitis: a meta-analysis of randomized, controlled trials. Int J Clin Exp Pathol.

[15] Zhang C, Wang Y, Fu W, Zhang W, Wang T, Qin H (2016). A meta-analysis on the effect of ulinastatin on serum levels of C-reactive protein, interleukin 6, and tumor necrosis factor alpha in Asian patients with acute pancreatitis. Genet Test Mol Biomarkers.

[16] Lagoo JY, D'Souza MC, Kartha A, Kutappa AM (2018). Role of ulinastatin, a trypsin inhibitor, in severe acute pancreatitis in critical care setting: a retrospective analysis. J Crit Care.

[17] Ranieri VM, Thompson BT, Barie PS (2012). Drotrecogin alfa (activated) in adults with septic shock. N Engl J Med.

[18] Salama C, Han J, Yau L (2021). Tocilizumab in patients hospitalized with Covid-19 pneumonia. N Engl J Med.

[19] Dolin HH, Papadimos TJ, Chen X, Pan ZK (2019). Characterization of pathogenic sepsis etiologies and patient profiles: a novel approach to triage and treatment. Microbiol Insights.

[20] Machado PR, Araújo MI, Carvalho L, Carvalho EM (2004). Immune response mechanisms to infections. An Bras Dermatol.

[21] Caille V, Bossi P, Grimaldi D, Vieillard-Baro A (2004). [Physiopathology of severe sepsis]. Presse Med.

[22] Lin GL, McGinley JP, Drysdale SB, Pollard AJ (2018). Epidemiology and immune pathogenesis of viral sepsis. Front Immunol.

[23] Templeton SP, Rivera A, Hube B, Jacobsen ID (2018). Editorial: immunity to human fungal pathogens: mechanisms of host recognition, protection, pathology, and fungal interference. Front Immunol.

[24] Han S, Mallampalli RK (2015). The acute respiratory distress syndrome: from mechanism to translation. J Immunol.

[25] Watanabe T, Kudo M, Strober W (2017). Immunopathogenesis of pancreatitis. Mucosal Immunol.

[26] Christaki E, Anyfanti P, Opal SM (2011). Immunomodulatory therapy for sepsis: an update. Expert Rev Anti Infect Ther.

[27] Marik PE (2018). Steroids for sepsis: yes, no or maybe. J Thorac Dis.

[28] Venkatesh B, Finfer S, Cohen J (2018). Adjunctive glucocorticoid therapy in patients with septic shock. N Engl J Med.

[29] Yao YY, Lin LL, Gu HY, Wu JY, Niu YM, Zhang C (2019). Are corticosteroids beneficial for sepsis and septic shock? Based on pooling analysis of 16 studies. Front Pharmacol.

[30] Rochwerg B, Oczkowski SJ, Siemieniuk RA (2018). Corticosteroids in sepsis: an updated systematic review and meta-analysis. Crit Care Med.

[31] Lamontagne F, Rochwerg B, Lytvyn L (2018). Corticosteroid therapy for sepsis: a clinical practice guideline. BMJ.

[32] Yang JW, Fan LC, Miao XY (2015). Corticosteroids for the treatment of human infection with influenza virus: a systematic review and meta-analysis. Clin Microbiol Infect.

[33] Arabi YM, Mandourah Y, Al-Hameed F (2018). Corticosteroid therapy for critically ill patients with Middle East respiratory syndrome. Am J Respir Crit Care Med.

[34] Stockman LJ, Bellamy R, Garner P (2006). SARS: systematic review of treatment effects. PLoS Med.

[35] Horby P, Lim WS, Emberson JR (2021). Dexamethasone in hospitalized patients with Covid-19. N Engl J Med.

[36] Rajapakse S, Rodrigo C, Maduranga S, Rajapakse AC (2014). Corticosteroids in the treatment of dengue shock syndrome. Infect Drug Resist.

[37] Tam DT, Ngoc TV, Tien NT (2012). Effects of short-course oral corticosteroid therapy in early dengue infection in Vietnamese patients: a randomized, placebo-controlled trial. Clin Infect Dis.

[38] Wiedow O, Meyer-Hoffert U (2005). Neutrophil serine proteases: potential key regulators of cell signalling during inflammation. J Intern Med.

[39] Wong WW (1998). ICE family proteases in inflammation and apoptosis. Agents Actions Suppl.

[40] Meng WT, Qing L, Li CZ, Zhang K, Yi HJ, Zhao XP, Xu WG (2020). Ulinastatin: a potential alternative to glucocorticoid in the treatment of severe decompression sickness. Front Physiol.

[41] Karnad DR, Bhadade R, Verma PK, Moulick ND, Daga MK, Chafekar ND, Iyer S (2014). Intravenous administration of ulinastatin (human urinary trypsin inhibitor) in severe sepsis: a multicenter randomized controlled study. Intensive Care Med.

[42] Yadav AK, Singh VK, Singh G, Singh V (2021). Outcome of ulinastatin vs metabolic resuscitation using ascorbic acid, thiamine and glucocorticoid in early treatment of sepsis-a randomised controlled trial. J Clin Diagn Res.

[43] Xu Q, Yan Q, Chen S (2018). Ulinastatin is effective in reducing mortality for critically ill patients with sepsis: a causal mediation analysis. Sci Rep.

[44] Mehta Y, Dixit SB, Zirpe KG, Ansari AS (2020). Cytokine storm in novel coronavirus disease (COVID-19): expert management considerations. Indian J Crit Care Med.

[45] Shanghai Clinical Treatment Expert Group for Corona Virus Disease 2019 (2020). Comprehensive treatment and management of corona virus disease 2019: expert consensus statement from Shanghai. Chinese J Infect Dis.

[46] Javeri Y, Rajani M, Juneja D (2015). Efficacy and safety of intravenous ulinastatin therapy in patients with severe dengue admitted in ICU. Indian J Crit Care Med.

[47] Lu Y, Chang J, Zhang M (2018). Identification and treatment of severe fever with thrombocytopenia syndrome. Int J Clin Exp Med.

[48] Xi XH, Lu SH, Mu YP (2012). Severe novel influenza A (H1N1) in Shanghai: clinical features, therapeutic management and risk factors for mortality. Infect Int.

[49] Zhu J, Liu Q, Cheng G, Zhang Z, Wang X (2020). A retrospective study of the effectiveness of ulinastatin in the treatment of sepsis. J Emerg Crit Care Med.

[50] Dominari A, Hathaway Iii D, Pandav K (2020). Thymosin alpha 1: a comprehensive review of the literature. World J Virol.

[51] Li C, Bo L, Liu Q, Jin F (2015). Thymosin alpha1 based immunomodulatory therapy for sepsis: a systematic review and meta-analysis. Int J Infect Dis.

[52] Liu F, Wang HM, Wang T, Zhang YM, Zhu X (2016). The efficacy of thymosin α1 as immunomodulatory treatment for sepsis: a systematic review of randomized controlled trials. BMC Infect Dis.

[53] Li Y, Li CS (2015). The therapeutic effects of thymosin α1 combined with human immunoglobulin (Ig) and bundles on severe sepsis: a retrospective study. Clin Lab.

[54] Sun Q, Xie J, Zheng R (2021). The effect of thymosin α1 on mortality of critical COVID-19 patients: a multicenter retrospective study. Int Immunopharmacol.

[55] Wu M, Ji JJ, Zhong L (2020). Thymosin α1 therapy in critically ill patients with COVID-19: a multicenter retrospective cohort study. Int Immunopharmacol.

[56] Liu Y, Pan Y, Hu Z (2020). Thymosin alpha 1 reduces the mortality of severe coronavirus disease 2019 by restoration of lymphocytopenia and reversion of exhausted T cells. Clin Infect Dis.

[57] Romani L, Bistoni F, Gaziano R (2004). Thymosin alpha 1 activates dendritic cells for antifungal Th1 resistance through toll-like receptor signaling. Blood.

[58] Wu J, Zhou L, Liu J (2013). The efficacy of thymosin alpha 1 for severe sepsis (ETASS): a multicenter, single-blind, randomized and controlled trial. Crit Care.

[59] Marik PE, Khangoora V, Rivera R, Hooper MH, Catravas J (2017). Hydrocortisone, vitamin C, and thiamine for the treatment of severe sepsis and septic shock: a retrospective before-after study. Chest.

[60] Fujii T, Luethi N, Young PJ (2020). Effect of vitamin C, hydrocortisone, and thiamine vs hydrocortisone alone on time alive and free of vasopressor support among patients with septic shock: the VITAMINS randomized clinical trial. JAMA.

[61] Vail EA, Wunsch H, Pinto R, Bosch NA, Walkey AJ, Lindenauer PK, Gershengorn HB (2020). Use of hydrocortisone, ascorbic acid, and thiamine in adults with septic shock. Am J Respir Crit Care Med.

[62] Wani SJ, Mufti SA, Jan RA (2020). Combination of vitamin C, thiamine and hydrocortisone added to standard treatment in the management of sepsis: results from an open label randomised controlled clinical trial and a review of the literature. Infect Dis (Lond).

[63] Shi R, Tie H (2020). Benefit of hydrocortisone, thiamine, and vitamin C for patients with sepsis or septic shock? Too early to draw conclusions. Crit Care.

[64] Laupland KB, Kirkpatrick AW, Delaney A (2007). Polyclonal intravenous immunoglobulin for the treatment of severe sepsis and septic shock in critically ill adults: a systematic review and meta-analysis. Crit Care Med.

[65] Yang Y, Yu X, Zhang F, Xia Y (2019). Evaluation of the effect of intravenous immunoglobulin dosing on mortality in patients with sepsis: a network meta-analysis. Clin Ther.

[66] Cui J, Wei X, Lv H, Li Y, Li P, Chen Z, Liu G (2019). The clinical efficacy of intravenous IgM-enriched immunoglobulin (pentaglobin) in sepsis or septic shock: a meta-analysis with trial sequential analysis. Ann Intensive Care.

[67] Nguyen AA, Habiballah SB, Platt CD, Geha RS, Chou JS, McDonald DR (2020). Immunoglobulins in the treatment of COVID-19 infection: proceed with caution!. Clin Immunol.

[68] Galeotti C, Kaveri SV, Bayry J (2020). Intravenous immunoglobulin immunotherapy for coronavirus disease-19 (COVID-19). Clin Transl Immunology.

[69] Cao W, Liu X, Hong K (2021). Corrigendum: high-dose intravenous immunoglobulin in severe coronavirus disease 2019: a multicenter retrospective study in China. Front Immunol.

[70] Gharebaghi N, Nejadrahim R, Mousavi SJ, Sadat-Ebrahimi SR, Hajizadeh R (2020). The use of intravenous immunoglobulin gamma for the treatment of severe coronavirus disease 2019: a randomized placebo-controlled double-blind clinical trial. BMC Infect Dis.

[71] Xie Y, Cao S, Dong H (2020). Effect of regular intravenous immunoglobulin therapy on prognosis of severe pneumonia in patients with COVID-19. J Infect.

[72] Mathias B, Szpila BE, Moore FA, Efron PA, Moldawer LL (2015). A review of GM-CSF therapy in sepsis. Medicine (Baltimore).

[73] Meisel C, Schefold JC, Pschowski R (2009). Granulocyte-macrophage colony-stimulating factor to reverse sepsis-associated immunosuppression: a double-blind, randomized, placebo-controlled multicenter trial. Am J Respir Crit Care Med.

[74] Hawchar F, László I, Öveges N, Trásy D, Ondrik Z, Molnar Z (2019). Extracorporeal cytokine adsorption in septic shock: a proof of concept randomized, controlled pilot study. J Crit Care.

[75] Mehta Y, Mehta C, Kumar A (2020). Experience with hemoadsorption (CytoSorb®) in the management of septic shock patients. World J Crit Care Med.

[76] Rizvi S, Danic M, Silver M, LaBond V (2021). Cytosorb filter: an adjunct for survival in the COVID-19 patient in cytokine storm? A case report. Heart Lung.

[77] Stockmann H, Keller T, Büttner S (2020). CytoResc - "CytoSorb" Rescue for critically ill patients undergoing the COVID-19 Cytokine Storm: a structured summary of a study protocol for a randomized controlled trial. Trials.

[78] Kes P (1998). Therapeutic plasma exchange in severe sepsis or septic shock. Acta Med Croatica.

[79] Stegmayr BG (1996). Plasma exchange in patients with septic shock including acute renal failure. Blood Purif.

[80] David S, Bode C, Putensen C, Welte T, Stahl K (2021). Adjuvant therapeutic plasma exchange in septic shock. Intensive Care Med.

[81] Khamis F, Al-Zakwani I, Al Hashmi S (2020). Therapeutic plasma exchange in adults with severe COVID-19 infection. Int J Infect Dis.

[82] Marano G, Vaglio S, Pupella S, Facco G, Catalano L, Liumbruno GM, Grazzini G (2016). Convalescent plasma: new evidence for an old therapeutic tool?. Blood Transfus.

[83] Hartman WR, Hess AS, Connor JP (2020). Hospitalized COVID-19 patients treated with convalescent plasma in a mid-size city in the Midwest. Transl Med Commun.

[84] Tremblay D, Seah C, Schneider T (2020). Convalescent plasma for the treatment of severe COVID-19 infection in cancer patients. Cancer Med.

[85] Agarwal A, Mukherjee A, Kumar G, Chatterjee P, Bhatnagar T, Malhotra P (2020). Convalescent plasma in the management of moderate covid-19 in adults in India: open label phase II multicentre randomised controlled trial (PLACID Trial). BMJ.

[86] Sun S, Liu D, Zhang H, Zhang X, Wan B (2019). Effect of different doses and time-courses of corticosteroid treatment in patients with acute respiratory distress syndrome: a meta-analysis. Exp Ther Med.

[87] Singh A, Pariti B, Mallayasamy SR, K V, Thunga G (2014). Role of corticosteroids use in ARDS: comparison of systematic review and meta-analysis. Value Health.

[88] Mammen MJ, Aryal K, Alhazzani W, Alexander PE (2020). Corticosteroids for patients with acute respiratory distress syndrome: a systematic review and meta-analysis of randomized trials. Pol Arch Intern Med.

[89] Lewis SR, Pritchard MW, Thomas CM, Smith AF (2019). Pharmacological agents for adults with acute respiratory distress syndrome. Cochrane Database Syst Rev.

[90] Yang ZG, Lei XL, Li XL (2017). Early application of low-dose glucocorticoid improves acute respiratory distress syndrome: a meta-analysis of randomized controlled trials. Exp Ther Med.

[91] van Paassen J, Vos JS, Hoekstra EM, Neumann KM, Boot PC, Arbous SM (2020). Corticosteroid use in COVID-19 patients: a systematic review and meta-analysis on clinical outcomes. Crit Care.

[92] Sterne JA, Murthy S, Diaz JV (2020). Association between administration of systemic corticosteroids and mortality among critically ill patients with COVID-19: a meta-analysis. JAMA.

[93] Mongardon N, Piagnerelli M, Grimaldi D, Perrot B, Lascarrou JB (2021). Impact of late administration of corticosteroids in COVID-19 ARDS. Intensive Care Med.

[94] Ji M, Chen T, Wang B (2018). Effects of ulinastatin combined with mechanical ventilation on oxygen metabolism, inflammation and stress response and antioxidant capacity of ARDS. Exp Ther Med.

[95] Fowler AA 3rd, Truwit JD, Hite RD (2019). Effect of vitamin C infusion on organ failure and biomarkers of inflammation and vascular injury in patients with sepsis and severe acute respiratory failure: the CITRIS-ALI randomized clinical trial. JAMA.

[96] Bharara A, Grossman C, Grinnan D (2016). Intravenous vitamin C administered as adjunctive therapy for recurrent acute respiratory distress syndrome. Case Rep Crit Care.

[97] Zhang M, Jativa DF (2018). Vitamin C supplementation in the critically ill: a systematic review and meta-analysis. SAGE Open Med.

[98] Chiscano-Camón L, Ruiz-Rodriguez JC, Ruiz-Sanmartin A, Roca O, Ferrer R (2020). Vitamin C levels in patients with SARS-CoV-2-associated acute respiratory distress syndrome. Crit Care.

[99] Zhang J, Rao X, Li Y (2021). Pilot trial of high-dose vitamin C in critically ill COVID-19 patients. Ann Intensive Care.

[100] Wadud N, Ahmed N, Shergill M (2020). Improved survival outcome in patients with SARS-COV-2 (COVID-19) ARDS with tocilizumab administration. Chest.

[101] Menzella F, Fontana M, Salvarani C (2020). Efficacy of tocilizumab in patients with COVID-19 ARDS undergoing noninvasive ventilation. Crit Care.

[102] Rosas IO, Bräu N, Waters M (2021). Tocilizumab in hospitalized patients with severe Covid-19 pneumonia. N Engl J Med.

[103] RECOVERY Collaborative Group (2021). Tocilizumab in patients admitted to hospital with COVID-19 (RECOVERY): a randomised, controlled, open-label, platform trial. Lancet.

[104] Guaraldi G, Meschiari M, Cozzi-Lepri A (2020). Tocilizumab in patients with severe COVID-19: a retrospective cohort study. Lancet Rheumatol.

[105] Atal S, Fatima Z, Balakrishnan S (2020). Approval of Itolizumab for COVID-19: a premature decision or need of the hour?. BioDrugs.

[106] Díaz Y, Ramos-Suzarte M, Martín Y (2020). Use of a humanized anti-CD6 monoclonal antibody (Itolizumab) in elderly patients with moderate COVID-19. Gerontology.

[107] Gore V, Kshirsagar DP, Bhat SM, Khatib KI, Mansukhani B (2021). Itolizumab treatment for cytokine release syndrome in moderate to severe acute respiratory distress syndrome due to COVID-19: clinical outcomes, a retrospective study. J Assoc Physicians India.

[108] Kumari P, Kumar A, Sinha C, Kumar A, Singh PK, Arun SK (2021). Off-label use of Itolizumab in patients with COVID-19 ARDS: our clinical experience in a dedicated COVID center. Indian J Crit Care Med.

[109] Kumar S, De Souza R, Nadkar M (2021). A two-arm, randomized, controlled, multi-centric, open-label phase-2 study to evaluate the efficacy and safety of Itolizumab in moderate to severe ARDS patients due to COVID-19. Expert Opin Biol Ther.

[110] Fredenburgh LE, Perrella MA, Barragan-Bradford D (2018). A phase I trial of low-dose inhaled carbon monoxide in sepsis-induced ARDS. JCI Insight.

[111] Horie S, McNicholas B, Rezoagli E (2020). Emerging pharmacological therapies for ARDS: COVID-19 and beyond. Intensive Care Med.

[112] Li J, Yang WJ, Huang LM, Tang CW (2014). Immunomodulatory therapies for acute pancreatitis. World J Gastroenterol.

[113] Xing B, Haifeng LI, Jin X, Wang H (2017). Effect of different doses of octreotide acetate on clinical efficacy of patients with severe acute pancreatitis. Chin J Integr Med.

[114] Pan Y, Fang H, Lu F (2017). Ulinastatin ameliorates tissue damage of severe acute pancreatitis through modulating regulatory T cells. J Inflamm (Lond).

[115] Abraham P, Rodriques J, Moulick N (2013). Efficacy and safety of intravenous ulinastatin versus placebo along with standard supportive care in subjects with mild or severe acute pancreatitis. J Assoc Physicians India.

[116] (2016). Indian Society of Gastroenterology. Indian J Gastroenterol.

[117] He HW, Zhang H (2020). The efficacy of different doses of ulinastatin in the treatment of severe acute pancreatitis. Ann Palliat Med.

[118] Guo H, Chen J, Suo D (2015). [Clinical efficacy and safety of ulinastatin plus octreotide for patients with severe acute pancreatitis]. Zhonghua Yi Xue Za Zhi.

[119] Zhu K, Wang JP, Su JG (2017). Prophylactic ulinastatin administration for preventing post-endoscopic retrograde cholangiopancreatography pancreatitis: a meta-analysis. Exp Ther Med.

[120] Elmunzer BJ, Scheiman JM, Lehman GA (2012). A randomized trial of rectal indomethacin to prevent post-ERCP pancreatitis. N Engl J Med.

[121] Petersen FC, Dahle UR, Nicolau B, Casals-Pascual C (2020). COVID-19: looking into the overlooked. Front Mol Biosci.

